# Recent Advances in Synthetic Bioelastomers

**DOI:** 10.3390/ijms10104223

**Published:** 2009-11-20

**Authors:** Rui Shi, Dafu Chen, Quanyong Liu, Yan Wu, Xiaochuan Xu, Liqun Zhang, Wei Tian

**Affiliations:** 1 Laboratory of Bone Tissue Engineering of Beijing Research Institute of Traumatology and Orthopaedics, Beijing 100035, China; E-Mails: sharell@126.com (R.S.); chendafugo@yahoo.com.cn (D.C.); 2 Beijing Key Laboratory on Preparation and Processing of Novel Polymer Materials at Beijing University of Chemical Technology, 100029, China; E-Mails: liu_quanyong@126.com (Q.L.); wuyan412@126.com (Y.W.); 3 Department of Spine Surgery of Beijing JiShuiTan Hospital, the Fourth Clinical Medical College of Peking University, Beijing 100035, China; E-Mails: xxcwyw@hotmail.com (X.X.)

**Keywords:** bioelastomer, biodegradable, biocompatible, polyurethanes, polyphosphazenes, poly(ether ester), poly(ε-caprolactone), poly(1, 3-trimethylene carbonate), poly(polyol sebacate)s, poly(diol-citrates), poly(ester amide)s

## Abstract

This article reviews the degradability of chemically synthesized bioelastomers, mainly designed for soft tissue repair. These bioelastomers involve biodegradable polyurethanes, polyphosphazenes, linear and crosslinked poly(ether/ester)s, poly(ε-caprolactone) copolymers, poly(1,3-trimethylene carbonate) and their copolymers, poly(polyol sebacate)s, poly(diol-citrates) and poly(ester amide)s. The *in vitro* and *in vivo* degradation mechanisms and impact factors influencing degradation behaviors are discussed. In addition, the molecular designs, synthesis methods, structure properties, mechanical properties, biocompatibility and potential applications of these bioelastomers were also presented.

## 1. Introduction

On the basis of the ASTM definition of ‘elastomer’, as well as the specific biological setting where bioelastomers are used, the ‘bioelastomer’ can be described as a substance with the following characteristics: good biocompatibility with neighboring/surrounding tissues; a glass transition temperatures (Tg) lower than body temperature (35–40 °C); the ability to return to at least 1.25-fold the original length after 1 min of release if stretched to 1.5-fold its original length for 1 min, maintaining the stretch stress in the range of 0.1–20 MPa. These biomaterials can be applied in the clinical fields of diagnosis, treatment, tissue repair or replacement, as well as tissue function enhancement.

The use of elastomers for biomedical applications goes back to the time when the rubber industry started. At that time, vulcanized natural rubber was used in medical devices. The commodity elastomers which happened to be used in biomedical fields and medical grades of elastomers certified for short-term physiological contact are not covered in this review.

Generally speaking, bioelastomers can be classified in two categories: 1) the elastomers suitable for long term physiological contact or implantation; 2) biodegradable elastomers for a determined time of physiological contact or implantation. With the development of tissue engineering, biodegradable elastomers have shown their strong advantages in clinical applications. As many tissues in the body have elastomeric properties, successful repair or replacement of these tissues will require the development of compliant biodegradable elastomeric scaffolds that can sustain and recover from multiple deformations without irritation to the surrounding tissue [1].

According to the product source, biodegradable elastomers can be classified into three categories: 1) naturally extracted and/or biosynthetic bioelastomers; protein- and/or peptide-based elastomers are included in this kind; 2) biosynthetic degradable polyesters; polyhydroxyalkanoates (PHAs) are the most important ones in this kind; 3) chemically synthesized biodegradable elastomers; these are what this review is about. For the natural (protein- and /or peptide-based) [2–4] and biosynthetic (PHAs) [5–11] readers are referred to the works by other researchers.

## 2. Requirements of Degradable Bioelastomers

In general, all biomedical elastomers must be of high purity, offer good processing performance, and optimal physical, chemical and mechanical properties. When they are implanted in the human body, adverse events such as thrombosis, cell injuries, plasma and protein degeneration, enzyme inactivation, electrolytes disturbances/imbalances, inflammation, carcinogenesis, intoxication and allergic reactions should not occur [12]. For biodegradable elastomers, the rate of degradation is one of the most important properties. For example, as a tissue engineering scaffold, the rate of the degradation should match that of tissue regeneration, with the material being completely degraded so as not to leave any reminding particles that may cause irritation or inflammation.

All biodegradable polymers contain hydrolysable bonds. For this reason, the most important mechanism of degradation for tissue engineering scaffolds is chemical degradation through hydrolysis or enzyme-catalyzed hydrolysis. To a lesser extent, oxidation can also be a degradation mechanism.

For most bioelastomers, passive hydrolysis is the dominant mechanism of degradation. There are several factors that influence the polymer biodegradability, including the type of chemical bond, polymer crystallinity, hydrophilicity, composition, pH value and temperature.

The type of chemical bond determines the rate of hydrolysis [8]. In general, anhydrides and orthoesters are the most hydrolysable, followed by esters and amides [13]. However, it should be noted that the rate of hydrolysis is greatly affected by catalysis. Hydrolysis can be thought of as a reaction between water and the labile bond. Consequently, the degradation rates for hydrophilic polymers are much greater than hydrophobic ones due to the ease of water absorbtion [8].

The most commonly parameter which is used to determine the degradation rate is the time dependent molecular weight. The polymer can be placed in an aqueous medium. Enzymes are always added to mimic the biological environment. In addition to molecular weight change, degradation may be assessed through mechanical property changes, weight loss, surface porosity, surface hydrophilicity (judged by contact angle), monomer or oligomer formation, or changes in pH value of the aqueous medium. Most of the time, a thorough understanding of the degradation mechanism should be obtained from all characteristics as a result of the sensitivities of these methods differ widely. For example, the mechanical properties can decrease dramatically even when the weight loss didn’t change so much.

The degradation procedure can be classified as surface degradation and bulk degradation [14]. The rate of the surface degradation should be easily predictable, because the degradation happened from the surface of the polymer, and the shape of the polymer is often kept well. This is particularly desirable for drug delivery applications, where the rate of drug release from the polymer can be directly related to the rate of degradation. The mechanical properties always have a linear relationship with degradation time. When bulk degradation happens, the weight is lost throughout the sample, but the original size of the samples could be kept for a long time. The bulk degradation maybe helpful to the drug bust release, and sometimes, tissue engineering scaffold.

The degradation rate can be measured *in vitro* and *in vivo*. For an accurate measurement, the *in vivo* degradation was often applied. The materials were implanted in the model animals. The changes of the biomaterial at the determine time interval was observed by X-ray diffraction, histological sections, etc.

## 3. Synthetic Bioelastomers

The types of biodegradable polymer, glass transition temperature, mechanical properties, approximate degradation time and the degradation products are summarized in Table 1.

### 3.1. Biodegradable Polyurethanes

The polyurethanes (PUs) have been the most popular group of biomaterials applied for biomedical devices for almost half a century, and this is because they provide great versatility in terms of tailoring their physical properties and biodegradation characteristics, as their properties can be tailored to range from biostable to rapidly degradable. Biostable PUs have been used commercially as biomedical implants since the 1960s, and improving the *in vivo* biostability of these materials has been investigated extensively [30]. In contrast to these biostable implants, biodegradable PUs are designed to undergo controlled biodegradation *in vivo* and promote new tissue ingrowths.

The term “polyurethane” in this thesis refers to segmented polyurethanes, which are sometimes described as poly(ester-urethanes) or poly(ether-urethanes). Phase separation always occurs in segmented polyurethanes and the two phases are generally referred to as the ‘hard segment’ composed of diisocyanate and chain extender, and the ‘soft segment’ due to a more amorphous macrodiol. The structure of the segmented PUs can be described by the following general formula [30]:

$$\text{P}—{\left[\text{D}\;{\left(\text{CD}\right)}_{\text{n}}—\text{P}\right]}_{\text{n}}$$

where the P is the polyol, D is the diisocyanate and C is the chain extender. The soft segment is an oligomeric macromonomer comprising a chain having a low transition temperature (below the room temperature) and terminated by hydroxyl groups. The chain extender is usually a small molecule with either hydroxyl or amine end groups. The diisocyanate is a low molecular weight compound that can react with either the polyol or chain extender. The combination of the chain and the diisocyanate components is referred to as the hard segment of the polymer.

#### 3.1.1. Biodegradable Soft Segments

Most biodegradable PU have soft segments composed of polyester or polyether macrodiols. The polyols used for biodegradable PUs synthesis are poly(ethylene oxide) (PEO), poly(propylene oxide) (PPO), poly(ε-caprolactone) (PCL), poly(d,l-lactide), poly(hydroxybutyrate) (P3HB and P4HB) and poly(glycolide). Woodhouse *et al*. synthesized a serious of PUs by using LDI and a novel amino acid ester chain extender in the hard segment, combined with PCL and/or PEO of different molecular weights as the soft segment [31–33].

According to their research, the PEO-based PUs were weak, tacky, amorphous materials, while the PCL-based PUs were strong and elastomeric. PEO is used to enhance degradation, while PCL provides crystallinity. It was demonstrated that the scaffolds prepared from PCL-b-PEO-b-PCL soft segments block copolymers supported faster hydrolytic degradation than that from the PCL soft segments PUs [34]. Soft segments were also designed by combining the PCL-b-PEO-b-PCL segments with other soft segments, such as α,ω-dihydroxyoligo [((*R*)-3-hydroxybutyrate-co-(*R*)-3-hydrocyalerate)-ethylene-glycol] to produce a family of PUs called DegraPol^®^, which was synthesized with either lysine methyl ester diisocyanate (LDI) or trimethylhexamethylene diisocyanate, along with the above soft segment [32,33,35]. Another example was the combination of the PCL-b-PEO-b-PCL segment with copolyester of adipic acid ethylene glycerol, diethylene glycol, and butanediol to produce another family called Diorez^®^ [36]. Other novel biodegradable soft segments were described in some references [37–45].

#### 3.1.2. Diisocyanates

A major limitation on the type of diisocyanate that can be used in biodegradable polymers for tissue engineering applications is the toxicity of the degradation products. As alternatives to 4,4’-methylenebis (phenylisocyanate) (MDI), lysine methyl ester diisocyanate (LDI) and 1,4-diisocyanatobutane (BDI) have been used to synthesize biomedical polyurethanes. Potential degradation products from these aliphatic diisocyanates are the amino acid lysine and the biological diamine putrescine. A lot of studies have reported that PUs biomaterials prepared from lysine-derived polyisocyanates biodegrade *in vitro* and *in vivo* to noncytotoxic decomposition products [46–48]. Some of the diisocyanate that have been applied in the synthesis of biodegradable polyurethanes are shown in Table 2.

#### 3.1.3. Biodegradable Chain Extenders

**Figure 1.**
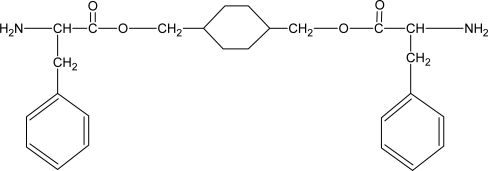
Structure of degradable chain extender used by Skarja and Woodhouse [16,51].

Some degradable chain extenders which incorporate amino acids have been previously developed. These degradable chain extenders are always diamines [50] (see Figure 1, for example).

**Figure 2.**
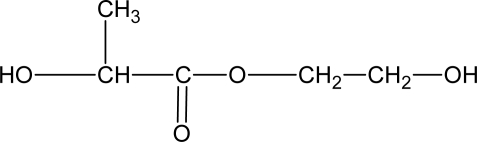
Structure of degradable chain extender based on lactic acid and ethylene glycol [16].

The chain extenders with phosphate ester linkages were degradable [52]. The phosphoester linkages in the backbone of the PU confer biodegradability on the polymer. However, no data related to the degradability of the PU was reported. A chain extender based on dl-lactic acid and ethylene glycol was reported [16]. These kinds of chain extenders were degradable and could accelerate hard segment degradation. The structure of the degradable chain extender is shown in Figure 2.

#### 3.1.4. Biodegradation Mechanism and Degradation Rate

Biodegradable PUs are designed to undergo enzymolysis or hydrolysis. The degradation mechanisms of hydrolysis were summarized by Guelcher [50,51]. The hydrolysis of ester linkages produces α-hydroxy acids, urethane and urea fragments with terminal acid groups. For lysine-derived polyisocyanates, hydrolysis of urethane linkages to lysine has been reported [37,44,45].

The degradation rate for biodegradable PUs is governed by many factors [52]. Most studies have suggested that the degradation rate was mainly controlled by polyester polyol composition [53,54]. PUs with amorphous soft segments have been observed to degrade more rapidly than those with semicrystalline soft segments, and those with hydrophilic soft segments have been reported to degrade more quickly than those with hydrophobic soft segments [55]. Most studies concluded that hydrolysis is the prevalent degradation pathway for *in vivo* degradation of poly(ester urethanes) [56–59]. Of all kinds of bonds present in poly(ester urethanes), the ester ones are the most susceptible to hydrolysis, while urethane and urea linkages are resistant to hydrolysis, and it has been reported that the latter are only enzymatically degraded [54,55,59,60]. All of these results suggest that the soft segment bonds will hydrolyse quicker than hard segment bonds, so the degradation rate of the biodegradable PUs is mainly controlled by their soft segments. The degradation of some polyester urethanes can also be controlled by the degree of crosslinking [61].

### 3.2. Biodegradable Polyphosphazenes

**Figure 3.**
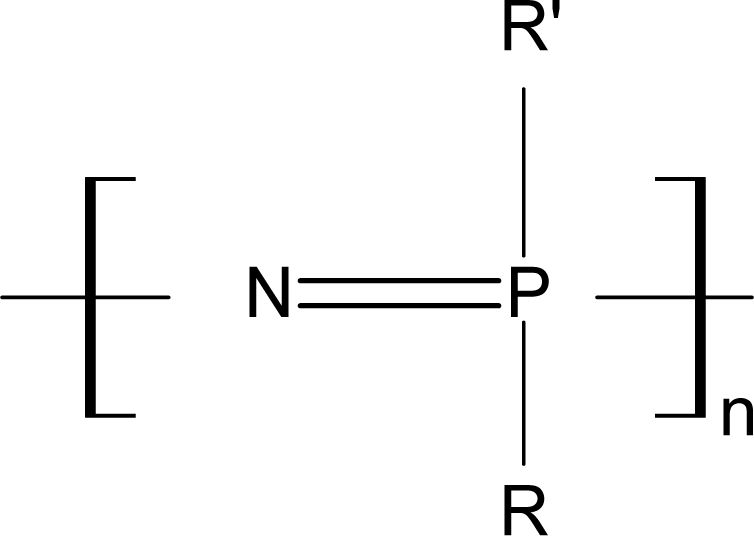
Structure of polyphosphazenes [62].

Polyphosphazenes (PNs) are a class of polymers of great potential for biomedical applications. These polymers consist of an inorganic backbone of alternating phosphorus and nitrogen atoms bearing side group substituents at the phosphorus atoms. Interest in these polymers is due to the fact that their properties are mainly dictated by the nature of the phosphorus substituents so that PNs for a wide range of applications can be obtained by the appropriate choice of the backbone derivatives. The degradation products of these degradable PNs are usually nontoxic, such as phosphates, ammonia and the corresponding side groups. The structure of the PNs is shown in Figure 3. The R and R’ in Figure 3 stand for organic groups or organometallic groups.

The physical properties of polyphosphazenes depend on the nature and the number of substituents. Because of the low rotational energy around the N-P bond (3.38 and 21.8 kJ/mol, respectively, for polyditrifluoroethoxy and polydiphenoxyphosphazene [63]) the structure of polyphosphazene has a high degree of freedom and a low glass transition temperature, so most PNs exhibit elastomeric properties. Small and unhindered substituents such as alkoxy groups give very low *T*_g_, below −60 °C (−105 °C for *n*-butyl). Aromatic rings give more stiffness, leading to a transition temperature of −34–0 °C with one ring, and 0–100 °C with two rings [64]. The freedom of movement of the chains can also be restricted by interatomic interactions. This is particularly true of primary amine substituted polymers which exhibit a *T*_g_ about 100 °C higher than polymers substituted with the corresponding alcohols. The P-N backbone flexibility can also make these polymers undergo structure changes in solid state [65]. The structure-properties relationship of some PNs are listed in Table 1 in Honarkar’s review [18].

The phosphorus-nitrogen backbone can be rendered hydrolytically unstable by substituting with appropriate side groups. Allcock and co-workers, who have explored extensively the field of polyphosphazenes [66–69], suggested that the biodegradable PNs generally comprise a hydrophilic substituent in a ratio corresponding to the required hydrophilicity, and an active principle either grafted or merely trapped in the polymeric network. Optionally a substituent capable of accelerating the hydrolysis of the chain by an assistance mechanism can also be added. The most frequently mentioned hydrophilic substituent is monomethylamine. polyethers [70], glucoses [71], and imidazole [72]. The presence of amino-acid esters among the substituents, in particular ethyl glycinate, helps the hydrolysis of the polyphosphazene backbone.

PNs can degrade by both surface and bulk erosion. As in the case of other hydrolytically sensitive polymers, the rate of degradation of PNs depends on several factors such as the lability of the bond, ease of water permeability, which in turn depends on the hydrophilicity or hydrophobicity of the matrix. Generally speaking, biodegradable PNs can be classified into two groups depending on the type of side group substituents: those substituted with amines and those substituted with activated alcohols [73,74]. The degradation models of these two kinds of biodegradable PNs were reviewed by Lakshmi [74].

#### 3.2.1. Aminated PNs

**Figure 4.**
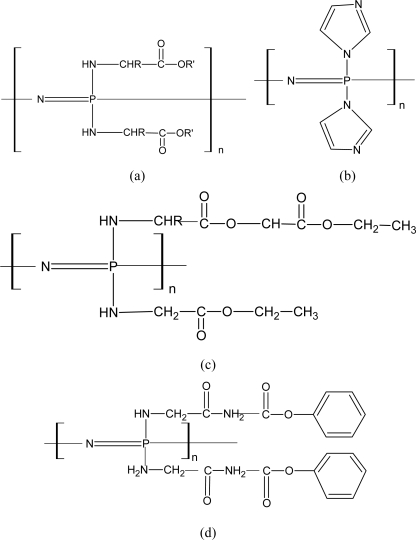
Structures of biodegradable aminated polyphosphazenes [74].

Aminated PNs comprise the largest and most extensively studied class of biodegradable PNs. Allcock and co-workers have synthesized a series of aminated PNs since 1966 [75–79]. The structures of these biodegradable aminated PNs are shown in Figure 4.

Among these PNs, the amino acid ester ones are perhaps the most extensively studied ones for biomedical applications. Figure 4(a) presents the imidazole-substituted PNs. The structure of Figure 4(b) was found to be the least stable among the aminated PNs synthesized. Figure 4(c) shows the structure incorporated side groups with hydrolytically sensitive ester functions [78]. Figure 4(d) gives an example of a highly pH-sensitive PN [79].

Two mechanistic pathways have been proposed for the hydrolytic instability of aminated PNs [74,80]. The degradation of these compounds can be triggered by the protonation of atoms in the skeleton or in the side groups. Protonation of skeletal nitrogen would lead directly to ring cleavage. This could ultimately lead to the conversion of the compound to ammonium ion, phosphoric acid and the free amine salt. protonation of side group nitrogen could take place, followed by nucleophilic attack by water at phosphorus, yielding a monohydroxycyclophosphazene, which, on further hydrolysis, would undergo ring cleavage and eventual degradation.

The ester functionality may be involved in the breakdown of the polyphosphazene skeleton via three different mechanisms. In the first, water would hydrolyze the ester unit to form the corresponding polymer-bound amino acid. The carboxylic acid unit could then attack a nearby phosphorus atom in the polymer chain. This species would then react further with water to release the amino acid and to form a hydrolytically unstable phosphazane, which would ultimately break down to phosphates and ammonia. In a second mechanism, the presence of water could facilitate an attack on the polymer backbone by the ester functionality itself. Water molecules could then react with the unstable phosphorus–ester bond. In a third mechanism, water would displace the amino acid esters from the phosphorus atoms to form a hydroxyphosphazene. This species could rearrange in the presence of water to a phosphazane. The phosphazane would then react with water to yield phosphates and ammonia. It was suggested that all three could occur simultaneously, although evidence for all three mechanisms was found [74,77,80].

The degradation rate can be modulated by some methods. It was shown by Allcock *et al.* [77] that the hydrolytic sensitivity of the polymers was found to decrease as the size of the ester groups increased. Similarly, the larger the group linked to the α-carbon atom of the amino acid residue, the more stable was the polymer to hydrolysis, so the degradation rate can also be modulated by incorporating suitable co-substituents along with the appropriate amino acid ester groups. The degradation rate was also affected by the pH. Ibim *et al.* [81]. found that the degradation rate was relatively much slower in neutral and basic solution (pH 7.4 and 10.0) than that in acidic solution. Results showed that more than 80% of the polymer depredated in 35 days at pH 2.0.

#### 3.2.2. Alkoxy-Substituted PNs

**Figure 5.**
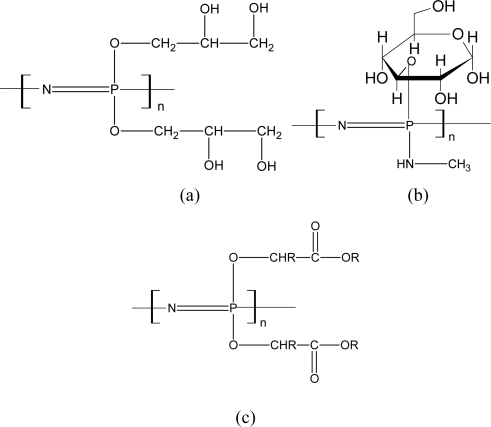
Structures of biodegradable Alkoxy-substituted polyphosphazenes [74].

The glyceryl-substituted polyphosphazene [Figure 5(a)] was first synthesized by Allcock and Kwon [82]. Structures of PNs substituted with glucosyl and methyl amino groups are shown in Figure 5(b) and esters of glycolic or lactic are also shown in Figure 5(c). They were reported to be hydrolytically labile [83,84]. The degradation products of the glucosyl and methyl amino substituents are presumed to be phosphate, glucose, ammonia and methylamine. The degradation process of glycolic or lactic PNs was described as: Water molecules could displace ethyl glycolato side units or hydrolyze the pre-ester function to the acid, and the pendent carboxylic acid could then induce skeletal degradation.

The *in vitro* and *in vivo* biocompatibility of biodegradable polyphosphazenes has been extensively investigated by Laurencin *et al.* [85–90]. Most of the amino acid ester polyphosphazenes elicited minimal to mild tissue responses when implanted subcutaniously in a rat model. Many of the amino acid ester polyphosphazenes have shown excellent osteocompatibility and have been investigated as matrices for bone tissue engineering [91]. Recently a polyphosphazene-self setting calcium phosphate composite cement system has been developed by taking advantage of the favorable interactions between polyphosphazenes side groups and calcium phosphate ceramics [92–95].

### 3.3. Network Polyesters Synthesized with Polyatomic Acids and Polyatomic Alcohols

#### 3.3.1. Poly(polyol sebacate)

Wang and coworkers [19] first reported the use of glycerol and sebacic monomers to produce a novel elastomeric material poly(glycerol sebacate) (PGS) for potential application in soft tissue engineering in 2002. Since glycerol is the basic building block of lipids, and sebacic acid is the natural metabolic intermediate in fatty acid oxidation [96,97], the degradation products of PGS are nontoxic. In addition, glycerol and copolymers containing sebacic acid have been approved for their application in medical applications by the U.S. FDA [98]. PGS demonstrated a favorable tissue response profile compared with PLGA, with significantly less inflammation and fibrosis and without detectable swelling during degradation.

Their simple synthesis process, adjustable mechanical and biodegradation properties, and good biocompatibility made the PGS elastomers excellent candidates for soft tissue engineering. They have already been investigated in tissue engineering applications [99–102].

**Figure 6.**
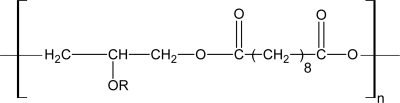
Structure of PGS [19].

PGS was synthesized by condensation polymerization. The structure of PGS is shown in Figure 6. To obtain the elastomers, they first synthesized a prepolymer and then poured an anhydrous 1,3-dioxolane solution of the prepolymer into a mold for curing and shaping under a high vacuum. The PGS obtained by Wang and coworkers was a kind of thermoset elastomer with a Young’s modulus of 0.282 ± 0.025 MPa, a tensile strain of at least 267 ± 59.4% and a tensile strength was at least 0.5 MPa. The mechanical properties of PGS were consistent with that of some common soft tissues. Liu and his coworkers [103–105], also investigated the effects of the molar ratio and the reaction conditions on the elasticity, strength, biodegradation and thermal processing abilities. The different molar ratios (glycerol/ sebacic acid) used in their work were 2/2, 2/2.5, 2/3, 2/3.5 and 2/4. They also prepared the thermoplastic PGS elastomer by a two-step method. Results demonstrated that the mechanical properties and degradation rates of products could be flexibly adjusted by the alteration of the molar ratio.

The degradation rate can be easily controlled by molar ratios and crosslinking density, which is related with the reaction temperature and time. The *in vitro* degradation was carried out in PBS at 37 °C under agitation. PGS lost 17 ± 6% of its mass, but during the same time *in vivo*, the copolymer was completely absorbed. The degradation rate *in vivo* is always quicker than that *in vitro* because of the enzymes originating from inflammatory cells or macrophages may attack the polymer bonds and catalyze the degradation process. The *in vivo* experiments showed that the mechanical properties (measured as compression modulus) decreased linearly and in parallel with the degradation of PGS, suggesting surface erosion as the mechanism of degradation [20]. Another important finding was that the shape of PGS was maintained during the degradation unlike that of PLGA. It was also proposed that PGS may perform well as a conduit material in peripheral nerve regeneration applications [99].

Beside the PGS, Bruggeman and his coworkers [106] have developed a family of thermoset poly-(polyol sebacate) (PPS) polymers. The material properties of PPS polymers can be tuned by altering the polyol monomer and the reacting stiochiometric ratio of sebacic acid. These thermoset networks exhibited tensile Young’s moduli ranging from 0.37 to 378 MPa, with maximum elongations at break from 10.90% to 205.16%, and T_g_ ranging from −7–46 °C.

Three general criteria led to the selection of polyols as monomers [106]: 1) non-toxic, 2) multi-functional to allow the formation of randomly crosslinked networks, as well as a wide range of crosslink densities, and 3) allow formation of hydrolysable esters in polycondensation polymerizations. xylitol, mannito and maltitol has been chosen as the monomers, corresponding to poly(xylitol sebacate) (PXS) 1:1 and PXS 1:2, poly(sorbitol sebacate) (PSS) 1:1 and PSS 1:2, poly(mannitol sebacate) (PMS) 1:1 and PMS 1:2, and poly(maltitol sebacate) (PMtS).

The *in vitro* degradation under physiological conditions was investigated. After 105 days of degradation, PXS 1:1 and 1:2 revealed a mass loss of 1.78% and 1.88%, respectively. PXS 1:1 did not reveal a similar mass loss profile as PSS 1:1 (15.66%) and PMS 1:1 (21.90%). PSS 1:1 and PMS 1:1 had degraded more than their corresponding 1:2 stiochiometries: PSS 1:2 had degraded 5.57 ± 1.00% of their original mass, and PMS 1:2 degraded 9.00% at this time. PMS 1:4 showed the least mass loss of 0.76%. In the *in vivo* degradation study, The PSS 1:1 elastomer appeared to have fully degraded after 12 weeks, without detectable trace despite repetitive sectioning of the implantation area. The *in vivo* degradation mechanism of PXS elastomers is dominated by surface erosion. The degradation rates *in vitro*, however, did not correspond to that *in vivo*.

#### 3.3.2. Poly(diol-citrates)

Citric acid was chosen as a multifunctional monomer to enable network formation. It is a nontoxic metabolic product of the body (Krebs or citric acid cycle), and has been approved by the FDA for its use in humans. It was found that the citric acid can be reacted with a variety of hydroxyl containing monomers at relatively mild conditions [21], meanwhile, it can also participate in hydrogen bonding interactions within a polyester network.

Yang and coworkers did the most efforts on the development of network polyester based on citric acid [21]. They investigated the reaction of citric acid with a series of aliphatic diols (from 3–16 carbon chains) and polyether diols such as polyethylene oxide, in which 1,8-octanediol (POC) and 1,10-decanediol (PDC) have been studied detailedly. The rationale behind these poly(diol-citrates) elastomers is [22]: 1) the use of non-toxic, readily available and inexpensive monomers. For example, citric acid is chosen as a multifunctional monomer that will be reacted via polycondensation with a difunctional monomers (diol) to form a cross-linked co-polymer; 2) incorporation of homogeneous biodegradable crosslinks to confer elasticity to the resulting material and leave behind some unreacted functional groups, which can be used for surface modifications; 3) the availability of various diols which provide flexibility to tune the mechanical and degradation characteristics of the resulting copolymer, and 4) the establishment of intermolecular hydrogen bonding interactions, which should contribute to the mechanical properties of elastomers.

**Scheme 1.**
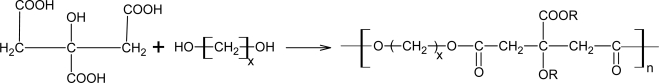
Synthesis of poly(diol citrates) [21].

The significant advantage of poly(diol citrates) when compared to existing biodegradable elastomers is that synthesis methods can be conducted under very mild conditions. Poly(diol citrates) were synthesized by reacting citric acid with various diols to form a covalent cross-linked network via a polycondensation reaction without using exogenous catalysts. The reaction scheme is shown in Scheme 1.

The mechanical properties, degradation and surface characteristics of poly(diol citrates) could be controlled by choosing different diols as well as by controlling synthesis conditions such as crosslinking temperature and time, vacuum and initial monomer molar ratio.

The tensile strength of poly(diol citrates) was as high as 11.15 MPa and Young’s modulus ranged from 1.60 to 13.98 MPa. Elongation was up to 502%. the tensile strength and Young’s modulus increased and the elongation at break decreased as the post-polymerization temperature and time increased [22], It was reported that POC is a strong elastomeric, biodegradable, and hydrophilic “cell-friendly” material [21]. The tensile strength was as high as 6.1 MPa and the Young’s moduli ranged from 0.92 to 16.4 MPa. The maximum elongation at break was 265% of initial length.

The degradation rate of POC is modulated by changing the post-polymerization temperature, reaction time, and the molar ratio of monomers. POC post-polymerized under mild conditions (low temperature, no vacuum) has a faster degradation rate than that post-polymerized under relatively tough conditions (high temperature, with vacuum). The totally degradation time of POC in PBS at 37 °C is about 6 months. Depending on implant location, the degradation rate may be faster *in vivo* due to enzymatic or cellular effects. Diols with decreasing number of methylene units result in increasing degradation rates.

Since the crosslink density is directly proportional to the strength but inversely proportional to the degradation rate, tailoring the properties of POC would be difficult if only the crosslink density can be varied. Increasing the molar ratio of citric acid increases the degradation rate of the copolymer without sacrificing its tensile strength. The introduction of *N*-methyldiethanolamine (MDEA) into the cross-linking net work significantly increased the degradation rate, while allowing the achievement of relative high tensile strength and Young’s modulus [107].

Composite materials of poly(diol citrates) were further investigated by Yang and his coworkers. They have prepared poly(diol citrates)/HA (hydroxyapatite) [107], poly(diol citrates)/chitosan [108], and poly(diol citrates)/PLA [108] composites with properties that are relevant to orthopedic and vascular tissue engineering applications.

In order to improve the mechanical properties of poly(diol citrates) and poly(glycerol sebacate), such as tensile strength and load bearing abilities, Dey and Yang *et al.* [61] developed a new class of biodegradable crosslinked urethane-dropd polyester elastomer. This class of polymers termed crosslinked urethane-doped polyesters (CUPEs) combines the advantages of fully elastic and biocompatible/hemocompatible crosslinked polyester networks with mechanically strong polyurethanes. Degradation studies were conducted in both phosphate buffered saline (PBS; pH 7.4) and NaOH solutions (0.01 M and 0.05 M). *In vitro* degradation studies showed that degradation rate was observed to be a function of the choice of diol, the isocyanate content, and the post-polymerization conditions. The polymers with higher isocyanate content exhibited faster degradation rates in both PBS and NaOH. Increasing the polymerization time made the CUPE polymers more resistant to degradation. Increasing the hydrophilicity of the diol used in the synthesis resulted in faster degradation rates as evidenced by the faster rate of degradation of the CUPE polymers containing poly(ethylene glycol) (PEG) in different ratios.

Besides the poly(polyol sebacate) and poly(diol-citrates), other multicomponent network polyester bioelastomers were also investigated. A series of network polyester bioelastomers–poly((1,2-propanediol-sebacate)-citrate)s (PPSCs), were synthesized by melt polycondensation of the polyfunctional monomer citric acid and oligomeric diol of 1,2-propanediolsebacate which was synthesized by controlled condensation reaction of difunctional monomers 1,2-propanediol and sebacic acid [109]. The mechanical properties, degradation and hydrophilicity of PPSC elastomers could be controlled by initial CA monomer molar ratio as well as by controlling the synthetic conditions such as cross-linking time. It was suggested that the synthesis of PPSC can also be carried out under mild conditions without addition of toxic catalysts, and they possess controllable mechanical and degradation properties which make them a good candidate for drug delivery and ther biomaterials.

The poly(diol citrates) have been designed into small-diameter blood vessel tissue engineering scaffold [110], nanoporous structure for drug-delivery reservoirs [111] and cartilage tissue engineering scaffold [112].

### 3.4. Biodegradable Poly(ether ester)

PEE copolymers were consisted of soft segments of polyethers and hard crystalline segments of polyesters. Depending on the polyether-polyester ratio, poly(ether ester) (PEE) copolymers exhibit a wide range of mechanical and biodegradation behaviors.

In all kinds of PEEs, poly(ethylene glycol)/poly(butylene terephthalate) (PEG/PBT) have been widely researched in the biomedical field. Segmented PEG/PBT multi-block copolymers are thermoplastic elastomers, which were both abbreviated as ‘PEG/PBT’ or ‘PEO/PBT’ in references. The ‘PEG/PBT’ form is adopted in this review. The composition of the block copolymers is abbreviated as a PEG b PBT c, in which a is the molecular weight of the PEG used, b is the weight percentage of PEG soft segments and c is the weight percentage of PBT hard segments.

**Figure 7.**
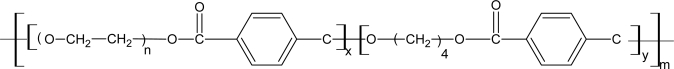
Structure of PolyActive^®^ [23].

In 1972, the potential medical application of PEG/PBT was accidentally discovered by Annis *et al.* [113]. When the PEG/PBT was implanted in animal body, they found that collagenic encystations was connected more closely with the tissue as increased of the PEG content. After the concept of tissue engineering formally proposed in 1988, the degradable PEEs elastomers attracted many researchers’ eyes. In the early 1990’s, Fakirov and and coworkers [114–124] prepared a series of PEG/PBT copolymers with different soft and hard segments content by using ester exchange method. Poly(ethylene glycol) (PEG 1000), 1,4-(butanediol) and dimethyl terephthalate were chosen as the raw materials, and titanate as the catalyst in their research. The effects of the initial ratio of the raw material on the physical and chemical properties were studied. At the same time, scholars in Holland such as Blitterswijk, Feijen, Bezemer, *et al*. widely studied the application of PEG/PBT copolymers in bone, cartilage and skin tissue engineering as well as in drug delivery system. Few years latter, the commercial product of PEG/PBT copolymers named PolyActive^®^ was firstly developed by the HC corporation in Holland. The structure of PolyActive^®^ was shown in Figure 7. After that the Isotis Biotechnology Corporation and some research organization (like Leiden University) worked together to develop tissue engineering scaffold by use of PolyActive^®^. The first study of them showed that the PolyActive^®^ could be used in bone replacement, artificial periosteum, wound dressing, artificial skin and drug control release carrier. Other clinical studies have been focused on the artificial tympanic membrane application [125,126] ventilation tubes, adhesion barrier [127], elastic bioactive coatings on load-bearing dental and hip implants [128] and also for wound healing purposes [129].

**Figure 8.**
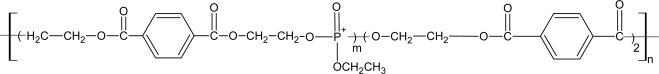
Structure of Politerefate^TM^ [23].

Another kind of commercial biomedical material named Politerefate^TM^ (Figure 8) has also been developed by inducing the phosphate groups into the PBT segments. Politerefate^TM^ has been widely investigated in nerve tissue engineering and control releasing drug delivery carrier [130,131].

A diverse family of customized polymers can be created by varying the amount and length of each of the two building blocks. The polymer matrix characteristics such as rate of controlled release, degradation, swelling and strength can be precisely controlled by the appropriate combination of the two copolymer segments. The segmented copolymers are phase separated, which is more obvious for polymers with high hard segment contents and polymers containing high molecular weight PEG. Tensile strengths were reported varies from 8 to 23 MPa and elongation at break changes from 500% to 1300%. Water-uptake ranged from 4% to as high as 210% [23]. Various *in vivo* studies have been performed to assess the biocompatibility of PEG/PBT copolymers upon implantation in soft and hard tissue [132–136]. Results can be concluded as that PEG/PBT copolymers did not induce any adverse affect on the surrounding tissue and thus showed a satisfactory biocompatibility.

The *in vitro* degradation of PEG/PBT copolymers occurs both by hydrolysis and oxidation, in which hydrolysis is the main degradation mechanism. In both cases, degradation is more rapid for copolymers with high PEG content [137]. Reed *et al*. investigated the degradation mechanics of PEG/PET copolymers whose structure is similar to that of the PEG/PBT. Although there are ether bonds, ester bonds among PET and ester bonds among the PEG/PET segments, only the last one was easy to hydrolyze [138]. Van Blitterswijk *et al.* [133] deduced that the degradation mechanics of PEG/PBT was similar to those of the PEG/PET. They pointed out that the ester bonds between the PEG and PBT segments hydrolyzed at first. The degradation products were PEG segments and low molecular PBT segments. The degradation rates were influenced by the composition, temperature, pH, enzyme content *et al*. The degradation rates increased as the PEG content, temperature, pH value increased, in which the component’ s content is the most impact factor on the degradation rates. Copolymers with high PEG contents degraded more rapidly than those with lower PEG contents. The initial degradation is restricted to the amorphous soft PEG segment. The fact that PEG was detected alone as major degradation product proved this assumption [139].

*In vivo* there are also two degradation pathways that are expected to take place. The first route involves hydrolysis of ester bonds in the PBT part or of ester bonds connecting PEG segments and terephthalate units. Besides of the hydrolysis, it has been suggested that *in vivo* degradation of the polymers with aliphatic ether groups involves phagocyte-derived oxidative degradation of PEG by a radical mechanism initiated at random along the chain [140]. Implantation of the copolymer devices provokes a foreign body response. During the implantation, specific activated cells such as macrophages release enzymes and superoxide anion radicals, which can combine with protons to form hydroperoxide radicals. So the oxidation degradation happened.

From the results of the accelerated hydrolysis experiments of 1000 PEG71PBT29 in PBS at 100 °C, the degradation products consisted of a fraction with high contents of PEG that was soluble in PBS and a PEG/PBT fraction that was insoluble at room temperature. Results from different *in vitro* and *in vivo* degradation experiments indicated that PEG/PBT degradation is a slow process and generates insoluble polymeric residues with high PBT contents. A part of the PBT fraction might remain in the body at late stages of degradation leading to incomplete degradation of PEG/PBT copolymers *in vivo* on the long term. However, the crystalline PBT fragments seem well tolerated by the surrounding tissues [141].

**Scheme 2.**
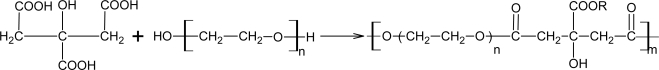
Reaction formula of condensing PEG and citric acid [142].

Except the lineal thermoplastic PEG/PBT elastomer composed with hard soft segments, the crosslinking network PEE was synthesized by PEG and polyprotic acid, such as citric acid to produce the poly(PEG-co-CA) (PEC) [142]. The pre-polymer synthesized by carrying out a condensation reaction between poly(ethylene glycol) and citric acid under atmospheric pressure, and then post-polymerized and cross-linked the pre-polymer in a mould at 120 °C. A relatively simple synthesis of PEC that can be carried out under mild conditions, especially without adding toxic catalysts or cross-linking reagents makes it a good candidate for biomaterials. The condensation reaction between PEG and citric acid is shown in Scheme 2. The degradation rate of the elastomer can be varied over a comparatively wide time range by controlling the cross-linked time and the initial monomer molar ratio.

### 3.5. Poly(ε-caprolactone) Copolymers with Glycolide or Lactide

Poly(α-hydroxy acids), mainly polylactide (PLA) and polyglycolide (PGA) homo- and copolymer are the first choice compared with other biodegradable polymers in preparation of biomaterials for tissue repair [143]. However, the stiffness and plastic deformation characteristics have limited their applications. Compared to the poly(α-hydroxy acids), PCL is a relatively flexible and biodegradable biomaterial. The safety of the PCL as several medical and drug delivery devices has been approved by FDA [144]. However, it seems the degradation rate of PCL is too slow for most tissue engineering applications. The total degradation of PCL may take two to three years. The copolymerizations of PCL with PLA or PGL can produce a novel material which will overcome the drawbacks of every single constituent, such as to provide better control over the degradation and mechanical properties without sacrificing biocompatibility. Specially, the copolymers are elastomeric which made themselves good candidate for soft tissue engineering [25], such as articular cartilage [145] and mechano-active vascular [146,147].

**Scheme 3.**
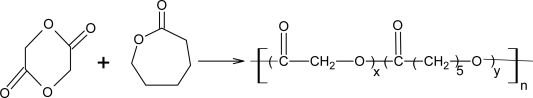
Synthesis of (PGCL) [24].

**Scheme 4.**
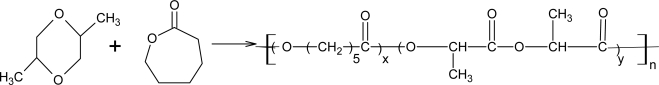
Synthesis of (PCLA) [24].

The poly(glycolide-co-caprolactone) (PGCL) and poly(lactide-co-caprolactone) (PCLA) copolymers were mainly synthesized by ring-opening polymerization. The reaction diagrams are shown in Scheme 3 and Scheme 4, respectively.

The degradation rate of PGCL or PCLA can be varied over a wide time range by tightly controlling the ratio between each kind of the monomers. Although the degradation products were not characterized, as degradation occurs through the hydrolysis of the ester bonds, the degradation products should contain short chain oligomers and the corresponding monomer hydroxyl acids (glycolic acid and 6-hydroxyhexanoic acid). All the copolymers degraded faster than each of the homopolymers. In Lee’s work, PGCL scaffolds (1:1 mole ratio) lost 3% of their initial mass after being incubated in PBS for two weeks and lost 50% after six weeks [25]. The result of Cohn’s work showed that the tensile strength of PCLA copolymers gradually dropped from their high initial levels (around 30–34 MPa) to 2–3 MPa after incubation in PBS for three months. Most of these copolymers remained flexible elastomers for a significant period of time in PBS [148]. In the *in vivo* degradation study, the implanted PCLA scaffolds displayed a slow degradation on time, whereas caprolactone units were degraded faster than lactide. This could be explained by the fact that amorphous regions composed of mainly CL moieties degraded earlier than hard domains where most of the LA units were located [149]. Based on the poly(α-hydroxy acids) and PLA, some other multiblock copolymers were synthesized, in which polylactide-co-poly(glycolide-co-caprolactone) was reported to be a biodegradable shape-memory polymer [150].

### 3.6. Poly(1,3-trimethylene carbonate) and Copolymers

Poly(1,3-trimethylene carbonate) (PTMC) is a rubbery and amorphous polymer [151]. It has already been investigated for the preparation of biodegradable elastomeric porous structures for soft tissue engineering scaffolds, specifically nerve and heart tissues [26,152]. Although PTMC is rubbery by itself, it is not an ideal candidate for implanted biomedical applications, because its degradation rates are difficult to predict. For example, although high molecular weight PTMC (M_w_: 500,000–600,000 g/mol) degrades very slowly (more than two years) when tested *in vitro* [153], it degrades within 3–4 weeks when implanted *in vivo*, the faster degradation most likely being due to enzymatic activity [27]. In order to obtain materials with suitable mechanical properties and degradation rates, TMC was copolymerized with either d,l-lactide (DLLA) or ε-caprolactone (CL) [26,152].

**Scheme 5.**
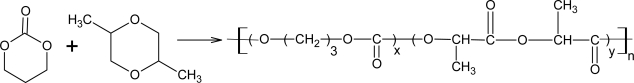
Synthesis of statistical poly(trimethylene carbonate-co-d,l-Lactide).

The properties of TMC–DLLA copolymers range from hard and brittle to rubbery and soft as the percentage of DLLA decreases. The copolymers with 20 to 50 mol% TMC are of most interesting in soft tissue engineering. The TMC-DLLA copolymers with 50 mol% TMC are highly flexible and strong with an elongation at break of 570% and an ultimate tensile strength of 10 MPa. The reaction of TMC with DLLA is shown in Scheme 5.

**Scheme 6.**
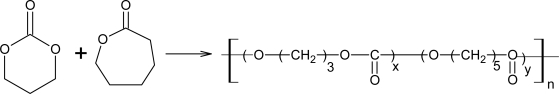
Synthesis of statistical poly(trimethylene carbonate-co-caprolactone).

TCM-CL based materials are all flexible. The strength increased as the CL content increased [26]. The reaction of TMC with CL is shown in Scheme 6.

*In vitro* degradation of TMC-DLLA was examined by incubating samples in PBS for up to two years [154]. The copolymers degraded much faster than the parent homopolymers, which is because the ester bonds are more labile to hydrolysis than the carbonate bonds. For P(d,l-LA), a continuously decrease of the mechanical properties was observed during the period of study, which could be related to the decrease in molecular weight (8% of its initial value after 60 weeks). In the case of PTMC, it did not show any significant decrease in molecular weight, and only a relatively small deterioration of the tensile strength (53%) was observed during this 60-week study.

It was observed that the degradation speed of poly(TMC-DLLA) (50:50) was the fastest of all samples. It degraded into the small pieces only after three months. Poly(TMC-DLLA) (20:80) became brittle, but still held its integrity after four months, while poly(TMC-DLLA) (79:21) lost its tensile strength after five months of degradation. Although the degradation products were not examined, one would expect that hydrolysis of the ester bonds will yield short chain oligomers and the original monomers.

The *in vitro* degradation rate of the TMC-CL based copolymers was much slower than that of the TMC-DLLA based copolymers. In accordance with the small decrease in molecular weight during the time of the study, no significant changes of water uptake, molecular weight distribution or mass loss were observed for the TMC-CL copolymers. The mechanical properties of TMC-CL copolymers as a function of degradation time decreased as the molecular weight decreased. All polymers maintained good mechanical performance, even after one year of degradation [154].

The degradation and the tissue response evoked by PTMC and copolymers of TMC with either 52 mol% DLLA or 89 mol% CL were evaluated *in vivo* by subcutaneous implantation of polymer films in rats for periods up to one year. PTMC specimens were extensively degraded after three weeks and, as confirmed by histology, totally resorbed in less than a year [27]. A fast linear decrease in thickness and mass without a change in molecular weight was observed. It is concluded that *in vivo*, PTMC is degraded via surface erosion involving cellular-mediated processes. The degradation of the copolymers was slower than that of PTMC, taking place via autocatalyzed bulk hydrolysis, preferentially of ester bonds. The TMC-DLLA copolymer degraded 20 times faster than the TMC-CL one. As the degradation of the TMC-CL copolymers was so slow, a mature fibrous capsule remained throughout the year. The TMC-DLLA copolymer became smaller and lost 96% of its total mass over the time course of this study. Histological results showed a normal inflammatory response and foreign body reaction. However, in the later stages of the study, the TMC-DLLA copolymers showed a second inflammatory reaction triggered by the cellular removal of residual polymer.

### 3.7. Poly(ester amide)s

Poly(ester amide)s (PEAs) are an interesting group of potentially biodegradable materials since due to the hydrogen bonding ability of the amide bonds and biodegradability imparted by the ester bonds, these co-polymers have good mechanical and thermal properties. Polyamides generally display better mechanical and thermal endurance than the corresponding polyesters, thanks to the formation of strong hydrogen bonding between the amide linkages of individual chains. Polyesters, on the other hand, are generally superior in flexibility, solubility, and hydrolytic susceptibility, and can thus be designed to degrade within a reasonable time-scale. The combination of polyester and polyamide moieties leaves us with tremendous opportunities of tuning the resulting properties to fit a desired profile.

PEAs can be generally classified into two major categories, depending on the sources that provide amide blocks: those synthesized from amino acids and those from non-amino acids source like aliphatic diamines. The non-amino acid based PEAs were initially reported by Barrows and others [155–157] and were synthesized from polyesterification of two types of amide diols, A radiolabel study of the degradation products in rats suggested that those non-amino acids based PEAs would degrade via the hydrolysis of their ester linkages, while their amide blocks remained stable.

To overcome the problem of limited biodegradability, amino acid units have been successfully incorporated into the backbone of poly(ester-amide)s to make it more attractive for enzymatic attack. The amino acid based PEAs, originally pioneered by Katsarava *et al*. [158–160], have however been made from naturally occurring amino acid building blocks and appear to be a very attractive new family of biodegradable biomaterials. They possess not only good biodegradability, but also good mechanical and processing properties, like thermal stability, tensile strength and elastic modulus. The biodegradability of different PEAs is one of the properties mostly investigated [161–167]. The hydrophobicity of the polymer molecules and their chain flexibility played an important role in the enzymatic catalyzed biodegradation of PEAs [163]. The PEAs have been prepared by polycondensation of ester-containing diamines with dicarboxylic acids or their derivatives, or by ring-opening polymerization of depsipeptides.

**Figure 9.**
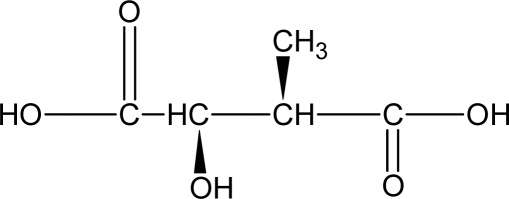
Structure of L-tartaric acid; (2*R*,3*R*)-(+)-2,3-dihydroxybutanedioic acid.

**Scheme 7.**
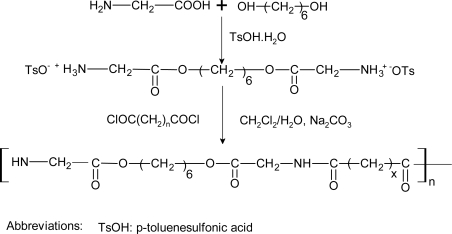
Synthesis of poly(ester amide)s derived from glycine, 1,6-hexanediol(H) and diacid [168].

The amino acids glycine, lysine and phenylalanine were separately copolymerized with AAc and 1,2-ethanediol to form poly(ester-amide)s of which the two latter were degradable by proteolitic enzymes, Similarly, poly(ester-amide)s were derived from 1,6-hexanediol, SA, and any of the amino acids alanine, phenylalanine, and glycine [169]. Glycine was also used for the copolymerization with 1,6-hexanediol and diacids with methylene group numbers ranging from 2 to 8 to prepare a series of aliphatic poly(ester-amide)s [92] with low molecular weights that were readily biodegraded by papain enzymes. The PEAs derived from glycine was synthesized according to the methodology outlined in Scheme 7.

(2*R*,3*R*)-l-tartaric acid (Figure 9) is an attractive monomer with two chiral carbon atoms [170]. Two families of PEAs were accordingly prepared from di-*o*-methyl-l-tartaric acid and succinic acid. The first one was synthesized by polycondensation in chloroform of ester trimers of 1,6-hexanediol and succinic anhydride with l-tartaric acid and succinic anhydride with l-tartaric acid and trimethylsilyl-activated 1,6-hexanediamine, resulting in random copolymers with varying contents of ester groups [171]. Hydrophilic and degradable PEAs were obtained. The modulus, strength, T_m_, and T_g_ decreases as the content of ester groups increased while the rate of hydrolytic degradation increased. Another approach to obtain stereoregular poly(ester-amide)s involved the polycondensation of diacids and optically active amino alcohols, such as 2-aminoethanol and leucinol [172].

A new class of synthetic, biodegradable elastomeric poly(ester amide)s composed of crosslinked networks based on the amino alcohol 1,3-diamino-2-hydroxypropane were developed by Bettinger *et al.* [173]. These crosslinked networks feature tensile Young’s modulus on the order of 1 MPa and reversable elongations up to 92%. These polymers exhibit *in vitro* and *in vivo* biocompatibility. These polymers have projected degradation half-lives up to 20 months *in vivo*.

The combination of ester and amide linkages in a polymer has been used for the preparation of tissue engineering scaffold and polymeric prodrugs. CAMEO® is a poly(ester amide) blend based on leucine or phenylalanine that is currently being developed for the site specific delivery of small hydrophobic drugs and peptides. Polyesters of malic acid have also been recognized as promising prodrug backbones. The pendant carboxylic groups of the poly(malic acid) main chain was covalently coupled to the anti-cancer drug adriamycin via amide or ester bonds. The cytotoxic activity of these prodrugs was evaluated *in vitro* [174].

## 4. Conclusions

Interest in engineering soft tissues, such as blood vessels, heart valves, nerve, bladder, cartilage, and tendons has prompted the development of novel bioelastomers, whose applications require or would benefit from the cyclic transfer of mechanical stimuli to cells or tissues. The biodegradable elastomers, which are similar to those of native tissue, have been paid more and more attention as the regenerative medicine developed. The bioelastomers discussed in this review are likely to become part of the available tools that used in tissue engineering to design novel scaffold to accelerate tissue regeneration.

This review mainly concerns biodegradable bioelastomers prepared by chemical synthesis methods. Biocompatible monomers were employed in these studies. The biggest advantage of this type of bioelastomers is the performance of materials can be regulated by changing the monomer composition and synthesis conditions. The results of all these studies clearly indicate that bioelastomers show excellent potential to be developed for a variety of tissue engineering applications.

## Figures and Tables

**Table 1.** Synthetic biodegradable bioelastomers.

**Synthetic Bioelastomers**	**Tg (°C)**	**TS (MPa)**	**EB(%)**	**Approx DT**		**Degradation Products**	**Referrences**
PUs	−116 – − 41	4–60	100–950	wide range	time	α-hydroxy acids, urethane, urea fragments, lysine (for lysine-derived polyisocyanates)	[15] [16]
PNs	− 105 −91	Wide range	Wide range	wide range	time	phosphate, ammonium salts, amino acids, and ethanol	[17] [18]
PGS^a^	−7 – 46	>0.5	>267	1		Glycerol, sebacate	[19,20]
POC^b^	−5 – 10	Up to 6.7	265 ± 10	variable		Octanediol, citric acid	[21]
PDC^c^	−5 – 10	Up to 3.14 ± 0.5	322 ± 20	variable		1,10-decanediol Citric acid	[22]
Poly(diol citrates)	− 5 10	Up to 11.2	Up to 502%	variable		Citric acid; polyols	[22]
PEG/PBT	–	8 to 23	500% to 1300%	wide range	time	PEG and PBT segment	[23,24]
PGCL^d^	–	<1	Up to 250	>1.5		Short chain oligomers; glycolic acid; 6-hydroxyhexanoic	[24,25],
TMC-DLLA (dry) 50:50 ^e^	11	10	570%	<11		Short chain oligomers; d,l-lactic acid; TMC monomers	[26–28]
TMC-DLLA (dry) 20:80^f^	33	51	7	<11		Short chain oligomers; d,l-lactic acid; TMC monomers	[26–28]
TMC-CL 10:90^g^	>−17	23	–	>24		Short chain oligomers; Caproic acid	[26–28]
PEAs	variable	variable	variable	wide range	time	Short chain oligomers; diamines,dicarboxylic acids	[29]

TS: tensile strength; T_g_: Glass transition temperature; EB: Elongation at Break; DT: Degradation Time (months);

a PGS: Poly(glycerol sebacate) 1:1 mole ratio polymerized for 77 h at 120 °C;

b POC: Poly(1,8-octanediol-co-citric acid) 1:1 mole ratio polymerized at temperatures ranging 80–120 °C for times ranging 1–4 days;

c PDC: Poly(1,10-decanediol-co-d,l-lactic acid) Mole% shown in parenthesis with polymerizations at 130 °C for 3 days;

e-f (TMC-DLLA): Poly(trimethylene carbonate-co-d,l -lactic acid) Mole% shown in parenthesis with polymerizations at 130 °C for 3 days.;

g Poly(trimethylene carbonate-co-caorolactone). Mole% shown in paraenthesis with polymerizations at 130 °C for 3 days.

**Table 2.** Diisocyanates in biodegradable PUs [49].

**Diisocyanates**	**Name and Abbreviation**
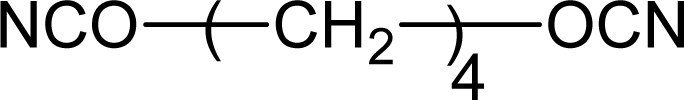	Butane diisocyanate (BDI)
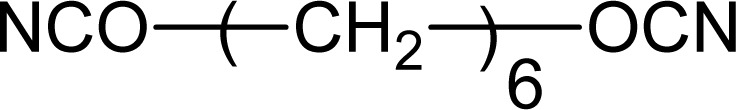	Hexamethylene diisocyanate (HDI)
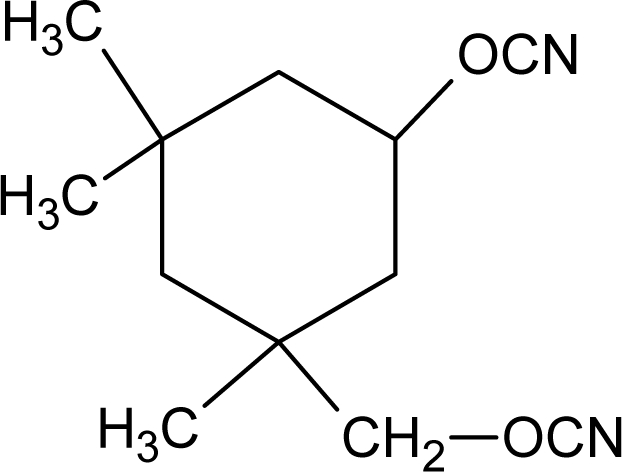	Isophorone diisocyanate (IPD)
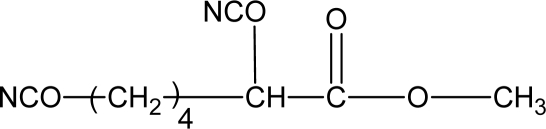	Lysine diisocyanate (LDI)
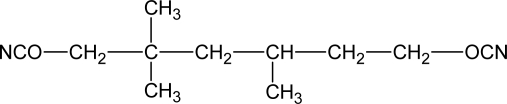	Trimethylhexamethylene diisocyanate (TMDI)
